# Utilization of bioelectrical impedance analysis for detection of lymphedema in breast Cancer survivors: a prospective cross sectional study

**DOI:** 10.1186/s12885-019-5840-9

**Published:** 2019-07-08

**Authors:** Sung Mook Lim, Yujin Han, Seung Il Kim, Hyung Seok Park

**Affiliations:** 1 Department of Surgery, Yonsei University College of Medicine, 50-1, Yonsei-ro, Seodaemun-gu, Seoul, 03722 Republic of Korea; 2 Department of Food and Nutrition, College of Human Ecology, Yonsei University, 50, Yonsei-ro, Seodaemun-gu, Seoul, 03722 Republic of Korea

**Keywords:** Breast cancer related lymphedema, Sentinel lymph node biopsy, Axillary lymph node dissection, Bioelectrical impedance, BMI

## Abstract

**Background:**

Breast cancer survivors are at risk of developing breast cancer-related lymphedema (BCRL) after surgical treatment, which may have a negative effect on quality of life. The purpose of this study was to investigate the clinical role of bioelectrical impedance analysis (BIA) and the relationship between the development of BCRL in breast cancer survivors who have undergone axillary surgery.

**Methods:**

A total of 228 patients with breast cancer were enrolled in the study between May 2016 and January 2017. BCRL was assessed by measuring the circumference of both arms at 15 cm below the acromion process and the olecranon process. Patients were classified as BCRL (*n* = 22) and non-BCRL (*n* = 206) based on the difference of the arm circumference of 2 cm. Data including lymphedema, anthropometry, BIA measurements, food frequency questionnaire, type of surgery, total number of dissected lymph nodes, and post-operative treatment were collected.

**Results:**

Of the breast cancer survivors, 10.4% had BCRL by the definition. The BCRL group contained 22 patients, while the non-BCRL group contained 206 patients. Compared to the non-BCRL group, the BCRL group had a higher body mass index, a larger percentage of ideal body weight, more dissected lymph nodes, and higher single frequency BIA (SFBIA) ratio (*P* = 0.027, *P* = 0.031, *P* < 0.001, and *P* < 0.001, respectively). The SFBIA ratio provided 63.64% sensitivity and 95.15% specificity in estimating the risk of BCRL.

**Conclusion:**

Our data provides evidence to support that the use of SFBIA ratio can serve as an alternative method to monitor and/or diagnose BCRL.

**Trial registration:**

This trial was retrospectively registered at Clinicaltrials.gov identifier (NCT03391206) on the 5 January 2018.

## Background

Lymphedema can be clinically diagnosed based on the swelling of limbs. However, definitive diagnosis of lymphedema is difficult, because most would suggest that lymphatic dysfunction imaged by lymphoscintigraphy or indocyanine green lymphography is required. The clinical diagnosis of lymphedema includes the observation that the bilateral difference in limb circumference is 2 cm or more [1], the difference of pre and post operation in volume of the limb is more than 200 ml [2], or the bilateral difference in volume of the limb change is 10% [3]. Because of differences in diagnostic criteria in these measurement methods, the definitive diagnosis is difficult. In addition, ultrasound, computed tomography, and magnetic resonance imaging are used to diagnose lymphedema. The ultrasound can measure volumetric and structural changes in the dermis, subcutaneous layer, and muscle, but information on the truncal anatomy of the lymphatic system can not be confirmed [4]. The computed tomography can detect thickening of the skin and subcutaneous compartment, and thickened perimuscular aponeurosis [4]. The magnetic resonance imaging can distinguish lymphedema, lipedema, and phlebedema, and can confirm the circumferential measurement edema, thickened dermis, and increased subcutaneous compartment [4, 5].

The arm circumference measurement is a commonly utilized clinical diagnosis method [6]. The circumference of both arms at 15 cm below the acromion process and the olecranon process is measured, and the circumference values of the affected arm and unaffected arm are compared [6, 7]. However, with this method there is no standardized reference point and low sensitivity. The lack of evidence-based diagnostic criteria to define lymphedema has presented tremendous challenges in terms of diagnosis. Therefore, defining criteria for the early detection and treatment of lymphedema is important [8].

Recently, several researchers have used bioelectrical impedance analysis (BIA) to diagnose lymphedema [9–14]. This method is highly sensitive, can be used as a basis to establish standardized criteria, and can be used to measure extracellular space [9, 15, 16]. Bioelectrical impedance predicts body composition using differences in electric conductivity upon sending a minute current through the human body [17, 18]. In several studies, the single frequency bioelectrical impedance analysis (SFBIA) of the two arms obtained using bioelectrical impedance measurements was expressed as the ratio of the values ​​of the operated and non-operated arms [10, 19]. However, this method has not been validated as a diagnostic tool. It is therefore necessary to study this method further to establish it as an efficient diagnostic means.

The purpose of this study is to determine diagnostic accuracy of bioelectrical impedance as a diagnostic method based on the presence of lymphedema compared with circumference measurements. In addition, the aim is to identify risk factors to help prevent lymphedema for breast cancer survivors.

## Methods

### Study design and subjects

This prospective study was conducted at Severance Hospital in Korea from May 2016 to January 2017 and involved female unilateral breast cancer survivors aged 20 or older who underwent surgery at least six months prior to selection. Patients with bilateral breast cancer, male breast cancer, recurrent breast cancer, previous ipsilateral axillary surgery, and previous radiotherapy were excluded. This study was approved by the Institutional Review Board of Severance Hospital (IRB Number: 4–2016-0149). All patients participated voluntarily in the study and provided written informed consent. All subjects underwent BIA, body measurements and semi-quantitative food frequency questionnaire for the Korean Genome Epidemiologic Study [20].

**Fig. 1:**
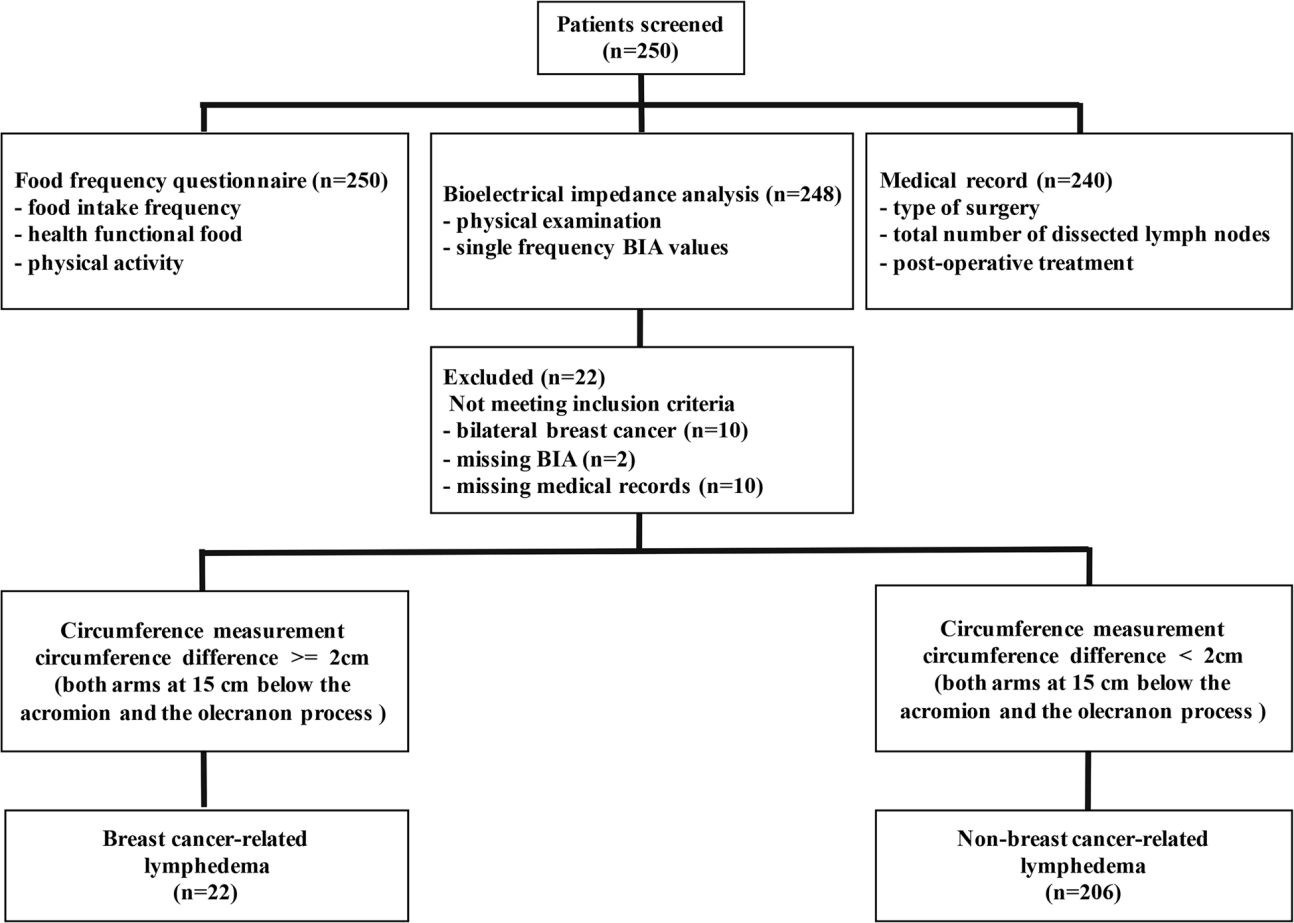
Flowchart illustrating the classification of the study participants in breast cancer survivors

A total of 250 patients were recruited. A total of 228 patients were finally enrolled in the study. (Clinicaltrials.gov identifier: NCT03391206). Of the patients who were excluded, 10 had bilateral breast cancer, 10 had poor medical records, and two did not have bioelectrical impedance measured (Fig. 1). The presence or absence of lymphedema was assessed in 228 patients based on circumference measurements [6]. Arm circumference measurements were examined using a method that was described in the previous study definition [21]. The circumference of both arms at 15 cm below the acromion and the olecranon process was measured, and the measured values of the affected and unaffected arm were compared. Patients were classified as BCRL group (*n* = 22) with a difference more than or equal to 2 cm and non-BCRL group (*n* = 206) with a difference less than 2 cm.

### Anthropometric parameter and body composition measurements

Body height was measured using an automatic extension meter. Body composition analysis was performed with an Inbody 720 composition analyzer (Biospace, Seoul, South Korea). Before assessment, participants were instructed to avoid excessive fluid intake, alcohol ingestion and heavy physical activity. Subjects were asked to remove anything metal and to stand barefoot on the metal footpads while loosely holding the handgrips. The results were automatically input into the system. Body weight, fat mass, body mass index (BMI), the waist-hip ratio (WHR), percent of ideal body weight (PIBW), extracellular water (ECW), total body water (TBW), and SFBIA were measured using BIA. SFBIA values were noted for both upper extremities at 1 kHz and 5 kHz. The SFBIA ratio was calculated as the ratio of the values of the affected to unaffected arms [14]. The SFBIA ratio was used to assess lymphedema.

BMI, PIBW, and SFBIA ratio were calculated as follows;

## BMI

BMI = body weight in kilograms / (height in meters)^2^.

## PIBW

PIBW = actual weight / ideal body weight^*^× 100.

^*^ideal body weight = (height in meters)^2^xideal BMI^#^.

^#^ideal BMI = female; 21 kg/m^2^, male; 20 kg/m^2^.

## SFBIA ratio

SFBIA ratio = unaffected SFBIA / affected SFBIA.

### Medical record collection

Clinicopathological information was obtained from the medical records of the participating women. Clinicopathological variables included surgery type (sentinel lymph node biopsy (SLNB) or axillary lymph node dissection (ALND)), number of dissected lymph nodes, and postoperative therapy such as chemotherapy, radiotherapy, hormone therapy, and target therapy.

### Statistical analysis

The variables used in this study were anthropometric values, the SFBIA value, the intake of nutrients, activity level, healthy functional foods, surgical methods, the number of removed lymph nodes, and treatment methods. The results are described as mean and standard deviation. The relationship between variables in the non-BCRL and BCRL groups was analyzed. The chi-squared test or Fishers exact test and independent sample t-test or Mann-Whitney U test were used for the analyses. The reported *p*-values are two-sided and were considered statistically significant at 0.05 or less. All data analyses were performed using IBM SPSS Statistics version 23.0 for Windows (IBM Corp., Armonk, NY, USA).

## Results

### Subject characteristics

Table 1 details the general characteristics of the study patients, who were classified according to axillary surgery type (SLNB or ALND) and the presence or absence of lymphedema (Non-BCRL or BCRL). No significant differences between the non-BCRL group and BCRL group were found in terms of age or physical activity level. The lymphedema of the operated site was significantly higher in the right side than in the left side. The number of lymph nodes removed was significantly higher in the BCRL than in the non-BCRL, but this difference was not observed when the subjects were divided according to surgery type (SLNB or ALND). The mean value of the acromion circumference difference was 2.69 ± 1.70 cm in the BCRL group. The arm circumference difference was larger in the lymphedema group with ALND than in the group with SLNB.

**Table 1:** General characteristics of the study population

	SLNB (*n* = 148)			ALND (*n* = 80)			Total (*n* = 228)		
	Non-BCRL	BCRL	*P* value^a^	Non-BCRL	BCRL	*P* value^b^	Non-BCRL	BCRL	*P* value^b^
	(*n* = 142)	(*n* = 6)		(*n* = 64)	(*n* = 16)		(*n* = 206)	(*n* = 22)	
Age (years)	53.2 ± 10.0	56.7 ± 15.0	0.556	59.1 ± 10.4	58.7 ± 6.7	0.124	52.8 ± 9.7	56.3 ± 12.0	0.121
Operated site									
Left	68 (47.9)	1 (16.7)	0.133	33 (51.6)	4 (25.0)	0.057	101 (49.0)	5 (22.7)	0.019
Right	74 (52.1)	5 (83.3)		31 (48.4)	12 (75.0)		105 (51.0)	17 (77.3)	
No. of dissected lymph nodes	4.2 ± 2.3	5.0 ± 2.5	0.507	15.7 ± 7.5	19.7 ± 7.4	0.057	7.7 ± 7.0	15.7 ± 9.2	0.001
Therapy^c^									
Chemotherapy	53	4		34	9		87	13	
Radiotherapy	88	4		47	15		135	19	
Hormonal therapy	113	4		44	12		157	16	
Target therapy	8	1		11	3		19	4	
Missing data	1	0		2	0		3	0	
Circumference difference^d^									
Acromion	0.57 ± 0.52	2.37 ± 0.59	0.000	0.71 ± 0.64	2.81 ± 1.97	0.001	0.58 ± 0.49	2.69 ± 1.70	0.000
Olecranon	0.58 ± 0.50	1.52 ± 1.02	0.010	0.54 ± 0.41	2.09 ± 2.10	0.010	0.55 ± 0.46	1.93 ± 1.867	0.002
Physical activity									
Inactive^e^	1 (0.7)	0 (0.0)	0.631	0 (0.0)	0 (0.0)	0.204	1 (0.5)	0 (0.0)	0.210
Sedentary^f^	23 (16.2)	1 (16.7)		11 (17.2)	5 (31.3)		34 (16.5)	6 (27.3)	
Active^g^	88 (62.0)	5 (83.3)		38 (59.4)	10 (62.5)		126 (61.2)	15 (68.2)	
Very active^h^	30 (21.1)	0 (0.0)		15 (23.4)	1 (6.3)		45 (21.8)	1 (4.5)	
Health supplement food^c^									
Vitamin & mineral agent	76	4		30	6		106	10	
Other dietary supplement	2	39		21	7		60	9	

*SLNB* sentinel lymph node biopsy, *ALND* axillary lymph node dissection, *non-BCRL* non-breast cancer-related lymphedema, *BCRL* Breast cancer-related lymphedema

Values are mean ± standard deviation or N (percentage)

^a^Differences between BCRL and Non-BCRL were tested by the Mann-Whitney *U* test and the Pearson’s chi-squared test

^b^Differences between BCRL and Non-BCRL were tested by the Student’s t-test and the Pearson’s chi-squared test

^c^Occasionally, one or more treatments were given to one person

^d^Circumference difference were calculated by the equation affected length minus the unaffected length

^e^Inactive: Limited physical activity (eg. inpatient)

^f^Sedentary: Most of the time is spent sitting in a static activity

^g^Active: Most of the time spent sitting, but lifestyle also includes standing work, commuting, buying things, housework, light exercise

^h^Very active: Strenuous work or highly active leisure

### Anthropometry and bioelectrical impedance data

The anthropometric data including body weight, fat mass, BMI, body fat percentage (BFP), WHR, PIBW, ECW/TBW, and SFBIA ratio were compared between the non-BCRL and BCRL groups for each surgery type (Table 2). BMI was significantly higher in the BCRL group (*P* = 0.027). Obesity-related factors including body weight, fat mass, BFP, PIBW, and BMI were significantly higher in the BCRL group than in the non-BCRL group for SLNB patients, while there were no significant differences among the ALND patients.

**Table 2:** Analysis of BCRL and non- BCRL values through bioelectrical impedance analysis

	SLNB			ALND			Total		
	(n = 148)			(n = 80)			(n = 228)		
	Non-BCRL	BCRL	*P* value^a^	Non-BCRL	BCRL	*P* value^b^	Non-BCRL	BCRL	*P* value^b^
	(n = 142)	(n = 6)		(n = 64)	(n = 16)		(n = 206)	(*n* = 22)	
BW (kg)	59.4 ± 10.6	72.4 ± 12.0	0.009	59.1 ± 10.4	58.7 ± 6.7	0.878	59.3 ± 10.5	62.4 ± 10.2	0.183
FFM (kg)	39.3 ± 4.5	43.8 ± 4.4	0.052	39.3 ± 4.7	38.4 ± 4.2	0.494	40.3 ± 14.6	39.9 ± 4.8	0.904
FM (kg)	20.1 ± 7.7	28.6 ± 8.0	0.016	19.8 ± 7.5	20.3 ± 4.8	0.816	20.0 ± 7.6	22.5 ± 6.8	0.131
BMI (kg/m^2^)^c^	23.8 ± 4.2	28.4 ± 4.5	0.011	23.6 ± 4.0	24.7 ± 2.7	0.275	23.7 ± 4.1	25.7 ± 3.6	0.027
BFP (%)	32.8 ± 7.2	38.8 ± 5.1	0.034	32.8 ± 6.5	34.2 ± 5.6	0.421	32.8 ± 7.0	35.5 ± 5.8	0.084
WHR	0.87 ± 0.06	0.89 ± 0.05	0.180	0.86 ± 0.05	0.89 ± 0.05	0.180	0.87 ± 0.06	0.89 ± 0.05	0.088
PIBW^d^	112.6 ± 20.0	134.7 ± 21.8	0.012	111.6 ± 19.0	116.9 ± 13.1	0.296	112.3 ± 19.6	121.7 ± 17.3	0.031
ECW/TBW	0.39 ± 0.01	0.39 ± 0.01	0.312	0.39 ± 0.01	0.39 ± 0.01	0.168	0.39 ± 0.01	0.39 ± 0.01	0.068

*SLNB* sentinel lymph node biopsy, *ALND* axillary lymph node dissection, non-*BCRL* non-breast cancer-related lymphedema, *BCRL* breast cancer-related lymphedema, *BW* body weight, *FFM* fat free mass, *FM* fat mass, *BMI* body mass index, *BFP* body fat percentage, *WHR* waist-Hip ratio, *PIBW* percent of ideal body weight, *ECW/TBW* extracellular water/total body water

Values are mean ± standard deviation

^*a*^*P* values of differences between means were calculated using the Mann-Whitney *U* test

^*b*^*P* values of differences between means were calculated using an independent sample *t*-test

^c^Body mass index(BMI) was calculated body weight in kilograms/(height in meters)^2^

^d^Percent of ideal body weight(PIBW) was determined by the equation actual weight (kg)/ideal body weight (kg) × 100

### Diagnosis of lymphedema using SFBIA ratio

Table 3 summarizes SFBIA ratios calculated from the ratio of the values of the operated and non-operated arms. The SFBIA values of the bioelectrical impedance were measured at 1 kHz and 5 kHz. The 1 kHz SFBIA ratio of the BCRL and non-BCRL groups was 1.145 ± 0.234 and 0.996 ± 0.039 (*p* < 0.001), respectively. The 5 kHz SFBIA ratio was significantly higher in the BCRL group than in the non-BCRL group (p < 0.001). Regarding the SFBIA ratio, a significant difference was observed between the non-BCRL and BCRL groups for ALND patients, but there was a tendency without significance for SLNB patients.

**Table 3:** SFBIA ratio^a^ of breast cancer survivors according to lymphedema

	SLNB			ALND			Total		
	(*n* = 148)			(*n* = 80)			(*n* = 228)		
	Non-BCRL	BCRL	*P* value^b^	Non-BCRL	BCRL	*P* value^c^	Non-BCRL	BCRL	*P* value^c^
	(n = 142)	(n = 6)		(n = 64)	(n = 16)		(n = 206)	(n = 22)	
1 kHz	0.991 ± 0.039	1.013 ± 0.045	0.183	1.006 ± 0.036	1.194 ± 0.257	< 0.001	0.996 ± 0.039	1.145 ± 0.234	< 0.001
5 kHz	0.990 ± 0.036	1.016 ± 0.037	0.075	1.004 ± 0.037	1.177 ± 0.250	< 0.001	0.994 ± 0.037	1.133 ± 0.225	< 0.001

*SLNB* sentinel lymph node biopsy, *ALND* axillary lymph node dissection, non-*BCRL* non-breast cancer-related lymphedema, *BCRL* breast cancer-related lymphedema, *SFBIA ratio* single-frequency bioelectrical impedance analysis ratio

Values are mean ± standard deviation

^a^Single-frequency bioelectrical impedance analysis(SFBIA) ratio of the affected to unaffected side were calculated

^*b*^*P* values of differences between means were calculated using the Mann-Whitney *U* test

^*c*^*P* values of differences between means were calculated using an independent sample *t*-test

**Fig. 2:**
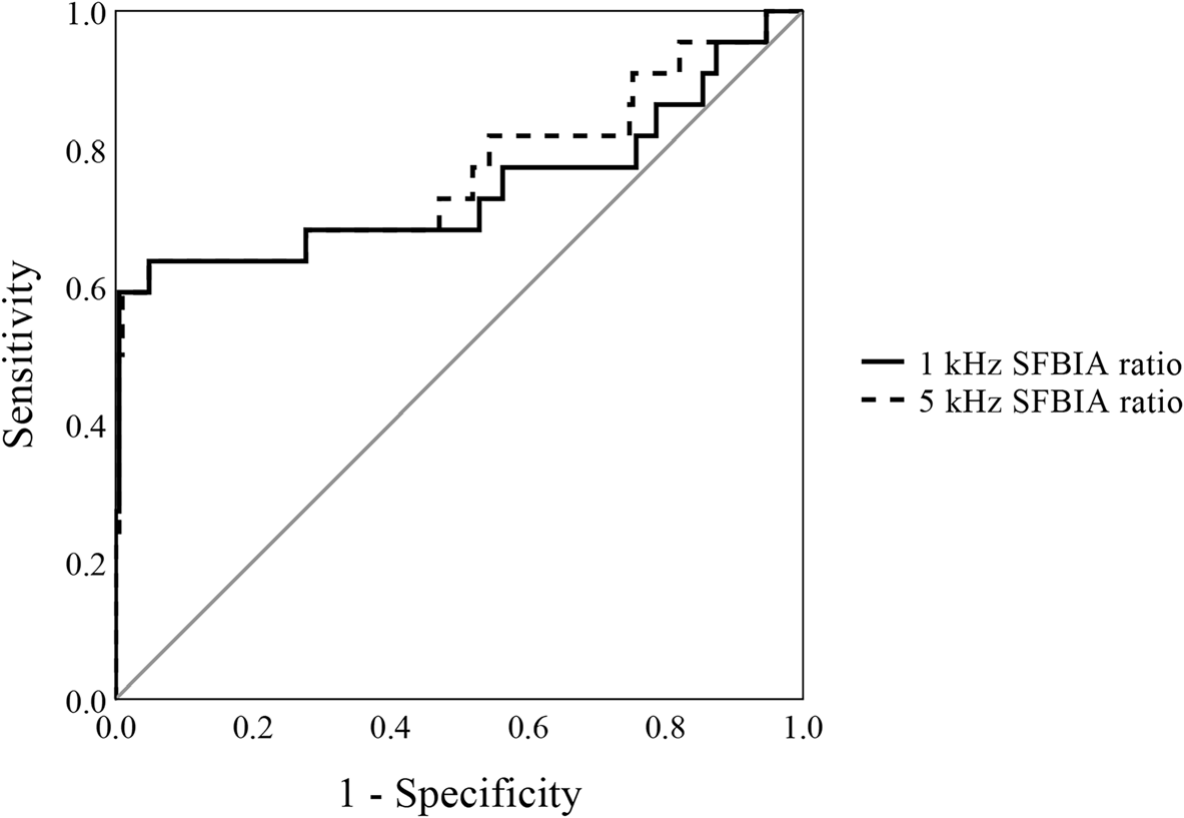
ROC curve of the 1 kHz and 5 kHz SFBIA ratio value. After the breast cancer surgery, the 1 kHz and 5 kHz SFBIA ratio of the survivors showed the good test performance to detect lymphedema with an area under the curve of 0.74 (95% CI: 0.59–0.89) and 0.77 (95% CI: 0.63–0.90), respectively. The 1 kHz and 5 kHz SFBIA ratio were effective predictors of post–BCRL(*p* = 0.000, *p* = 0.000)

Receiver operating characteristic curve (ROC) analyses were performed in order to evaluate diagnostic ability of SFBIA ratio using the1 kHz and 5 kHz SFBIA ratios. Figure 2 shows the ROC curve in 1 kHz and 5 kHz. Our SFBIA ratio cut-off value are 1.049 and 1.047, respectively. Area under the curve of 5 kHz SFBIA ratio was higher than that of 1 kHz (5 kHz; 0.77, 1 kHz; 0.74). The 5 kHz SFBIA ratio showed better performance as a diagnostic tool compared to 1 kHz SFBIA ratio with an area under the curve of 0.77 (95% CI: 0.63–0.90). The 5 kHz SFBIA ratio values were used as criteria for determining the occurrence of lymphedema in each patient. The analysis of diagnostic accuracy of the 5 kHz SFBIA showed specificity of 95.15%, sensitivity of 63.64%, positive predictive value of 58.33%, and negative predictive value of 96.08%.

## Discussion

Early prevention and detection of postoperative lymphedema complications in breast cancer patients is important for quality of life. Our data provided evidence to support the use of the SFBIA ratio by BIA in lymphedema in breast cancer survivors. In addition, right axillary surgery was suggested to be a risk factor associated with lymphedema. Lymphedema was more common in patients with the right axillary procedure than those with the left. This may be due to the fact that right handed people are more common and have more axillary activity on the right side. Additionally, the number of dissected lymph nodes [22–25], obesity [23, 24, 26, 27], and surgery type [22, 24, 25] were risk factors for lymphedema. It was concordant with previous study [22–27].

In this study, BCRL was determined based on more than a 2 cm difference in the circumference, as measured 15 cm below the olecranon, or acromion process, of the arm not affected by the operation relative to the circumference of the arm on the same side as the operation [7, 28, 29]. Limitations include potential errors in the measurements and the fact that diagnosis is not possible until clinical symptoms are seen. More precise techniques including ultrasound, computed tomography, magnetic resonance imaging, lymphoscintigraphy and other volumetric measurement can enhance the diagnosis of lymphedema. Further studies that evaluate the comparison these techniques and BIA are needed.

BIA is designed to measure edema as an extremely small electrical current passes through extracellular fluid [9]. This technique distinguishes extracellular fluid from total limb volume [9, 30]. In the presence of lymphedema, the SFBIA ratio [10] is related to the accumulation of extracellular fluid [31]. Our results show that the SFBIA ratio is larger at 5 kHz than at 1 kHz. BIA had good performance in terms of specificity (95.15%) and negative predictive value (96.08%). A diagnostic tool with a high specificity is more useful for ‘judging’ a disease when a person is positive and the negative predictive value can be used that the probability of not having disease given a negative diagnosis [32]. These values indicate that BIA can be used as a method of monitoring and diagnosing lymphedema. Previous studies have reported that early surveillance for risk of lymphedema using bioimpedance spectroscopy with early intervention with compression garments can reduce the incidence of more advanced lymphedema [11, 12]. Our cut-off value of the SFBIA ratio for diagnosing lymphedema should be validated in further studies. The usefulness of early detection through the SFBIA ratio is necessary to be evaluated as well.

Well-known risk factors for lymphedema include the surgery type [22, 24, 25] and the number of dissected lymph nodes [22–25]. Similarly, our study also demonstrated that the higher the number of lymph nodes removed in the ALND subjects, the higher the incidence of lymphedema. However, these were not observed for the SLNB because the number of lymph nodes removed is too small to affect the risk of lymphedema in these subjects. Overall, on average our SLNB subjects had less than 5 lymph nodes removed whereas the ALND had 16 to 20 lymph nodes removed. Thus, surgery type associated with high lymph node removal is likely to increase the risk of lymphedema as reported previously [22–25].

Obesity-related indicators in breast cancer patients increase the risk of developing lymphedema complications [26, 33, 34]. Our data investigated the relationship between the incidence of lymphedema and the variables related to anthropometric measurements and type of surgery. The BMI and PIBW of the subjects were significantly higher in the presence of lymphedema. In particular, body fat percentage, BMI, and PIBW were significantly different in patients who underwent SLNB. These findings suggest that the occurrence of lymphedema is associated with obesity and that patients who undergo SLNB procedures should pay attention to maintaining normal weight. There was no significant difference of obesity-related indicators between BCRL and non-BCRL in the patients with ALND. Our results were different from the studies conducted on Westerners. The association between BMI and lymphedema volume in patients with ALND was observed [35, 36]. The difference between ours and previous studies may be due to difference of study population, a race, culture, lifestyle, and dietary differences between Westerners and Asians. Further research is needed to understand the factors behind these differences.

The lymphedema of our subjects was confirmed by the difference in limb circumference of 2 cm of both sides. This confirmed the lymphedema of the arm, but not the lymphedema that appeared at other sites. Therefore, the diagnosis of more detailed lymphedema will establish an accurate standard of the SFBIA ratio. Nevertheless, our study confirmed the cut-off value of the SFBIA ratio for the determination of lymphedema through 228 subjects and confirmed the sensitivity and specificity. Our findings have shown the possibility of SFBIA ratio as a useful tool for the diagnosis and management of lymphedema in breast cancer survivors. In addition, we found that there was a significant correlation between lymphedema and obesity in patients who underwent SLNB, but not in patients who underwent ALND.

## Conclusion

The SFBIA ratio obtained using BIA can be an alternative method for monitoring and/or diagnosing BCRL. The BIA had a sensitivity of 63.64% and a specificity of 95.15% in predicting BCRL. In addition, number of dissected lymph nodes, operation site, surgery type, obesity, and the SFBIA ratio are significantly associated with the occurrence of lymphedema.

## Data Availability

The datasets generated and/or analyzed during the current study are not publicly available due to domestic regulation that prohibits opening clinical data of patients but they are available from the corresponding author upon reasonable request.

## Abbreviations

ALND: Axillary lymph node dissection
BCRL: Breast cancer-related lymphedema
BFP: Body fat percentage
BIA: Bioelectrical impedance analysis
BMI: Body mass index
ECW: Extracellular water
PIBW: Percent of ideal body weight
ROC: Receiver operating characteristic
SFBIA: Single frequency bioelectrical impedance analysis
SLNB: Sentinel lymph node biopsy
TBW: Total body water
WHR: Waist-hip ratio
